# The role of amyloid beta clearance in cerebral amyloid angiopathy: more potential therapeutic targets

**DOI:** 10.1186/s40035-017-0091-7

**Published:** 2017-08-17

**Authors:** Xue-mei Qi, Jian-fang Ma

**Affiliations:** Department of Neurology & Institute of Neurology, Ruijin Hospital Affiliated to Shanghai Jiaotong University School of Medicine, Shanghai, 200025 China

**Keywords:** Cerebral amyloid angiopathy, Alzheimer’s disease, Amyloid β-protein, Clearance

## Abstract

Cerebral amyloid angiopathy (CAA) is characterized by the deposition of amyloid β-protein (Aβ) in the leptomeningeal and cortical blood vessels, which is an age-dependent risk factor for intracerebral hemorrhage (ICH), ischemic stroke and contributes to cerebrovascular dysfunction leading to cognitive impairment. However clinical prevention and treatment of the disease is very difficult because of its occult onset and severity of the symptoms. In recent years, many anti-amyloid β immunotherapies have not demonstrated clinical efficacy in subjects with Alzheimer’s disease (AD), and the failure may be due to the deposition of Aβ in the cerebrovascular export pathway resulting in further damage to blood vessels and aggravating CAA. So decreased clearance of Aβ in blood vessels plays a crucial role in the development of CAA and AD, and identification of the molecular pathways involved will provide new targets for treatment. In this review, we mainly describe the mechanisms of Aβ clearance through vessels, especially in terms of some proteins and receptors involved in this process.

## Background

Cerebral amyloid angiopathy (CAA) is the second reason (after hypertension) causing cerebral hemorrhage in the elderly, accounting for 15–40% of non-traumatic cerebral hemorrhage in the elderly with a mortality of 30–50% [1]. Occasionally, CAA can be presented as cerebral ischemic attack, cognitive dysfunction and cerebral vasculitis [2, 3]. In addition, CAA is commonly found in Alzheimer’s disease (AD) and nearly 80% of AD patients are accompanied by CAA [4].

The main pathological feature of CAA is the deposition of amyloid β-protein (Aβ) in the tunica media and adventitia of the arterioles and/or capillaries in the cerebral cortex and leptomeninges [5]. Aβ deposited in AD senile plaques is mainly Aβ_42_, however it’s usually Aβ_40_ that deposited in the vascular wall of CAA. Sporadic CAA is commonly classified into two categories based on the presence or absence of Aβ on capillaries: CAA-type 1 is defined if the deposition of Aβ on cortical capillaries beside leptomeningeal, cortical arteries and arterioles, and CAA -type 2, not involving cortical capillaries.

In physiological conditions, human brain can produce Aβ without abnormal accumulation because Aβ can be moved out through several mechanisms quickly and effectively: (1) uptake and degradation by glial cells; (2) degradation by proteolytic enzymes; (3) clearance through blood brain barrier (BBB); (4) interstitial fluid bulk-flow clearance (perivascular drainage or clearance by glymphatic pathway); (5) complement-related clearance. One proposed pathogenesis of CAA is that inefficient Aβ clearance leads to abnormal Aβ accumulation in the brain and vessels, causing CAA in aged brain. Based on this assumption, several therapeutic interventions have been tried in CAA animal models by enhancing Aβ clearance and drainage systems. For example, experimental gene therapy to up-regulate neprilysin in the brains of aged Tg2576 mice has been reported to reduce Aβ levels [6]. Promoting perivascular drainage can facilitate Aβ_40_ clearance and improve cognitive deficits in Tg-SwDI mice [7]. Administrating ponezumab, an anti- Aβ_40_ selective antibody, to transgenic mice led to a reduction of Aβ deposition and an improvement of vessel function [8]. How to make these basic neuroscience progresses into clinical effective therapies requires more comprehensive understanding of mechanisms involving Aβ clearance under pathological conditions. This review will focus on recent findings of Aβ clearance system and try to discuss the potential interventional targets for future CAA treatment.

### Enzyme degradation

Aβ-degrading enzymes including neprilysin, insulin-degrading enzymes (IDE), angiotensin-converting enzyme (ACE), cathepsin, etc., play an important role in Aβ clearance and have a protective role in CAA by reducing the damage of Aβ to vascular smooth muscle cells. A previous review have summarized their crucial role in AD and CAA, and the up-regulation of cerebral Aβ degrading enzyme has potential therapeutic effect on AD [9]. Here we focus on their role in CAA pathology. For example the expression of vascular neprilysin reduced in CAA patients and the decrease was more obvious in Apoε4 carriers [10, 11]. Gene polymorphisms of neprilysin has also been reported to be related to sporadic CAA and disease severity [12]. Both vitro and vivo studies have demonstrated that up-regulation of neprilysin could reduce Aβ concentration and be beneficial to AD [13, 14]. A recent study suggests that neprilysin activity is suppressed directly or indirectly by dual-specificity tyrosine phosphorylation-regulated kinase 1A (DYRK1A), so DYRK1A inhibition may also be a promising therapeutic target for AD through up-regulating neprilysin [15]. Another Aβ degrading enzyme IDE isolated from human brain microvessels has been shown to be capable of degrading Aβ40, and the IDE protein levels was increasing in AD patients with CAA, however its degrading activity was reduced in CAA microvessels [16]. And for ACE, it’s has been shown to cleave Aβ40 at the site Asp(7)-Ser(8). And the degradation products Aβ-(1-7) and Aβ-(8-40) peptides were less aggregated or cytotoxic [17]. The activity of ACE-1 was increased in AD patients and in moderate to severe CAA vessel-associated ACE-1 levels were higher [18]. Further study found that ACE variants are related to ICH recurrence in CAA, possibly by regulating ACE expression [19]. Up-regulation of Aβ degrading enzyme has potential therapeutic effect on AD and further studies are needed to assess their role in treatment for CAA pathology.

### The transcytosis of Aβ across BBB

Blood brain barrier (BBB) refers to plasma and brain barrier composed of cerebral capillary wall and glial cells, as well as a barrier between plasma and cerebral spinal fluid (CSF) composed of choroid plexus named blood–cerebrospinal fluid barrier (BCSFB). BBB can limit the transport of polar molecules into the brain, but the necessary nutrients such as glucose, amino acids, and vitamins can permeate through BBB mediated by receptors on the vascular endothelium. BBB also allows the transport of larger molecules, such as neuroactive peptides and proteins, and plays an important role in the regulation of brain Aβ concentration.

**Fig. 1:**
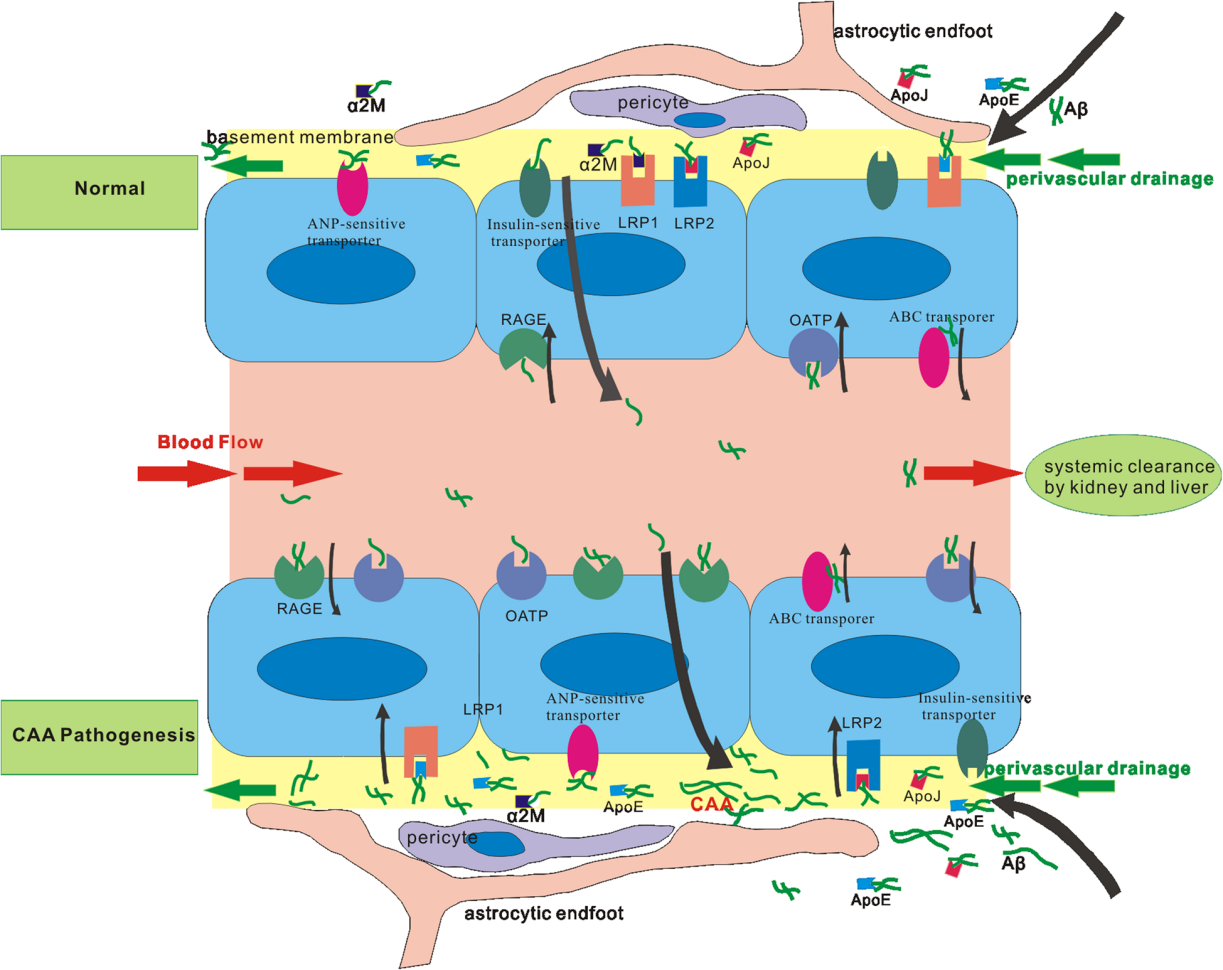
Aβ can be transported bi-directionally through BBB by multiple receptors. In normal conditions the transportation of Aβ can be mediated by multiple receptors in endothelium. After binding to ApoE or α2M (α2-microglobulin) Aβ can be transported by LRP1 or it can be transported by LRP2 after binding to ApoJ (clusterin). Some other receptors also mediate Aβ efflux, such as ABC transporter, insulin-sensitive transporter and ANP-sensitive transporter. There’s only little Aβ influx mediated by RAGE and OATP. In addition Aβ can be transported to perivascular spaces and eliminated through perivascular drainage. In CAA pathological condition, there’s a change in the transporter profile of the BBB, with the efflux receptors decreasing and the influx receptors increasing, leading to the decrease of Aβ clearance and its deposition on the vessel wall. Consequently components changes of cerebrovascular basement membrane as well as the weakness of perivascular drainage results in the aggregation of Aβ in blood vessels aggregating CAA

Aβ can be transported bi-directionally through BBB by multiple receptors in the vascular endothelium (Fig. 1). Receptors involved transporting peripheral Aβ into the brain consist of advanced glycation end products (RAGE), organic anion transporting polypeptides (OATP) such as Oatp1a4. The receptors mediating Aβ clearance from the brain to the peripheral circulating system include low-density lipoprotein receptor family (LDLR family), ATP-binding cassette transporters (ABC transporters), insulin-sensitive transporter, natriuretic peptide receptor C (Npr-C). These receptors regulate the influx and efflux of brain Aβ and maintain the balance of Aβ under normal condition. So any dysfunction of this transportation system can disturb the balance of Aβ distribution and lead to Aβ aggregation in the vessels which contributes to CAA formation in the brain.

### Receptors mediating Aβ influx

#### Advanced glycation end products (RAGE)

RAGE belongs to immunoglobulin receptor superfamilies and can interact with several ligands including soluble Aβ. The expression of RAGE was increased in capillary of CAA patients and APP transgenic mice, suggesting the association of RAGE with Aβ aggregation in the capillary [20]. Further study found that in 3xTg-AD transgenic mice, the exogenous pathogenic gene could up-regulate RAGE expression in endothelium cell [21]. RAGE can mediates the transport of Aβ_40_ or Aβ_42_ across BBB into brain, resulting in endothelial cell oxidative stress and expression of proinflammatory cytokines and NF-κB through redox-dependent activation of Ras-ERK1/2 pathway, p38 MAP (p38), Cdc42/Rac pathway and SAPK/JNK kinase pathways [22], which finally leads to cell apoptosis, inflammatory response and vascular dysfunction. And the inhibition of RAGE-ligand interaction reduces aggregation of Aβ in brain parenchyma in transgenic mouse [23]. Using an in vitro BBB model, Candela et al. [24] found that specific competitive inhibitor against RAGE could decrease the apical-to-basolateral transport of Aβ_40_ and Aβ_42_ significantly, which was a caveolae-dependent process through endothelial cells. And recently, a research observed that 1,25-dihydroxyvitamin D3 (1,25(OH)2D3) increased the efflux of Aβ40 from brain to blood through up-regulating LRP1 and down-regulating RAGE [25].

Several drugs targeting on RAGE have been tried for treating AD and CAA. Inhibition of RAGE-ligand interaction by using soluble RAGE (sRAGE) or anti-RAGE antibody can suppress the accumulation of Aβ in brain parenchyma in transgenic mouse models [23, 26]. Specific inhibitor against RAGE can alleviate amyloid deposition as well as improve cognitive function in APP transgenic mouse [27]. TransTech Pharma, Inc. discovered TTP488, which acts as an antagonist of RAGE-RAGE ligand interaction. Chronic oral dosing of this drug in AD transgenic mouse resulted in a reduction of amyloid deposition in the brain and an improvement of behavioral performance and in phase 2 clinical trial in mild to moderate AD, TTP488 has achieved positive results [28].

#### Organic anion transporting polypeptides (OATP)

Members of the OATP family, OATP1A2/SLCO1A2 (Oatp1a4/Slco1a4 in mice) and OATP14 (Oatp14/Slc21a14 in mice), are expressed on the luminal and abluminal sides of brain capillary endothelial cells. Statins are effective substrates for OATP and the uptake of statins in the liver is mainly dependent on OATP transport. As one kind of the cholesterol reducing drugs, statin has been shown helpful to prevent AD and the protective effect is probably not only related to their ability to reduce cholesterol levels, but also some other mechanisms may also be involved [29], such as competitively binding OATP with Aβ. Do et al. [30] found that rosuvastatin and taurocholate, two established Oatp1a4 substrates, decreased Aβ influx, while its inhibitor L-thyroxine increased Aβ influx. So Oatp1a4 might play an important role in the Aβ clearance from brain. More studies are needed to reveal the specific function between Oatp1a4 and Aβ, as well as whether its inhibitor L-thyroxine contributes to the CAA and AD in pathological process.

### Receptors mediate Aβ efflux

#### ATP-binding cassette transporters (ABC transporters)

ABC transporter is a member of the biggest protein superfamilies, existing in all living organism from microorganism to human. The human ABC transporter is encoded by 49 genes and is divided into A to G 7 subfamilies based on sequence homology and functional similarity. ABC transporters utilize ATP to provide energy for transport of polar and non-polar molecules across cell membrane, which plays an important role in physiological conditions, and its function defects can lead to serious genetic diseases. The transporters are highly expressed in barrier structure (blood-brain barrier, blood-testis barrier, blood-placental barrier), excretory organs (liver, kidney) and absorption organs (small intestine, colon). Some members of the transporters in ABC transporter subfamilies B, C, G can discharge metabolic wastes, exogenous substances and many drugs from the central nervous system to the blood. Among them the most studied substances include multidrug resistance proteins (MDR1), ATP-binding cassette B1 (ABCB1) or P-glycoprotein (P-gp), multidrug resistance-associated protein (MRPs), ATP-binding cassette G2 (ABCG2), ATP-binding cassette G1 (ABCG1) or breast cancer resistance protein (BCRP) and ATP-binding cassette G4 (ABCG4). Recent studies have found that ABC transporters are involved in Aβ clearance.

ABCG2 is highly expressed in CAA and AD transgenic mouse brain and can inhibit the influx of Aβ_40_ across BBB. In vitro study, inflammatory mediators released by Aβ-activated microglia enhance the expression of ABCG2 in vascular endothelial cell [31]. However Carrano et al. [32] observed that the expression of ABCG2 and ABCB1 decreased in capillary of CAA patients, but was not changed in AD and normal controls. And using a vitro BBB model, they further found that Aβ_42_ oligomers or co-incubating Aβ_42_ with clusterin (apolipoprotein J) down-regulated the expression of ABCB1 in the vascular endothelium without any change of ABCG2, suggesting the special function of ABCB1 in capillary CAA, and a recent study observed that Aβ40 could mediate the ubiquitination, internalization and proteasome-dependent degradation of ABCB1 in isolated rat brain capillaries, which indicates that the ubiquitin-proteasome pathway is associated with the lower ABCB1 protein levels in vascular endothelium exposing to Aβ40 [33]. 3xTg-AD transgenic mouse studies demonstrated that the expression of Aβ transporter protein differs in different disease stages. Although the exogenous APP gene can up-regulate the expression of influx transporter RAGE and down-regulate the expression of efflux transporter LRP1, mice can counteract this increased net influx by up-regulating ABCG4、ABCG2、ABCB1, and maintain the balance of Aβ influx and efflux in BBB [21]. Molecules in CSF including Aβ can also be removed via Aβ transporters at the BCSFB, such as ABCB1, LRP1, LRP2 [34]. During aging, there’s a significant alterations in Aβ transporter profile expressed at BCSFB with Aβ efflux transporters increased and Aβ influx transporters decreased [35, 36]. So BBB and BCSFB is a dynamic barrier, which can adapt to different pathological conditions by changing its transporter profile. More researches are required to elucidate the role of ABCG2 in the pathogenesis of CAA and AD.

#### Low-density lipoprotein receptor family (LDLR family)

LDLR family includes at least 10 kinds of receptors, including LDLR, VLDLR, LRP1, LRP1B, LRP2, LRP3, LRP4, LRP5, LRP6 and LRP8. Previous studies revealed that LDLR family was of crucial importance for the development of the nervous system, aging and pathogenesis of AD [37]. The most well-known function of this receptor family is receptor-mediated endocytosis.

LRP1 expression decreases with aging especially for AD patients [38]. Along the progression of AD, hypoxia occurs and stimulates the overexpression of the serum response factor (SRF) and myocardin in cerebral vascular smooth muscle cells. SRF and myocardin can further activate sterol regulatory element binding protein-2, which could down-regulate LRP1 [39]. Many LRP1 ligands co-deposite with Aβ in senile plaques and are involved in Aβ clearance (Fig. 1), such as apoE, α2-microglobulin (α2M), lactoferrin, urokinase-type plasminogen activator, tissue-type plasminogen activator [40]. Studies have suggested that ApoE4 can block the clearance of soluble Aβ from brain by LRP1 [41], leading to the increasing Aβ deposition in the vascular wall and pathological changes of CAA in APP and ApoE4 double transgenic mice. ApoE4-expressing mice has an elevated ratio of Aβ40:42 in brain extracellular pools and a lower Aβ40:42 ratio in CSF, which suggests that ApoE4 leads to the altered transport and clearance of Aβ proteins by LDLR in different brain compartments [42], a possible explanation for the lower Aβ levels in CSF of AD patients with cortical microbleeds [43]. The lipidization level of different ApoE isoforms is not the same. The low lipidization level ApoE4 promotes Aβ deposition, while the higher lipidization level ApoE2 promotes Aβ clearance by LRP1 [44]. While ApoE2 genotype is protective for AD, it’s related to intracerebral hemorrhage in CAA patients [45].

The cell surface LRP1 has been shown not only related to Aβ cell uptake and the lysosomal degradation of Aβ in vascular smooth muscle cells [46], but also mediating the Aβ efflux through BBB and further elimination by the liver, spleen and kidney [47]. And LRP1 may influence the phagocytosis or macropinocytosis of Aβ, because it has been respected to control cytoskeleton architectures through focal adhesion kinase (FAK)/paxillin and/or phosphoinositide 3-kinase (PI3K)/extracellular signal-regulated kinase (ERK) pathways [48, 49]. Recently Steffen E. Storck and colleges developed transgenic mouse models that allow for specific deletion of LRP1 within brain endothelial cells. The selective deletion of brain LRP1 in 5xFAD transgenic mouse can reduce plasma Aβ levels and elevate soluble brain Aβ, resulting in aggravated spatial learning and memory deficits [50]. So LRP1 plays an important role in Aβ clearance in BBB via various ligands. Apart from this LRP1 is a multifunctional receptor that can regulate several signaling pathways by binding to other receptors to play a role in the inflammation of atherosclerosis, cancer, and nervous system injury [51]. It can also regulate gene expression through the intracellular domain [49].

LRP2, also named as megalin, is the biggest receptor of the LDLR family, expressed in a variety of absorption epithelial cells such as small intestinal brush border cells and mainly expressed in endothelial cells and choroid plexus in the brain. LRP2 can recognize variety of ligands with different structure and functions including lipoprotein (apoE, clusterin), vitamin-binding proteins, hormones, neurotrophic factors and so on. Many studies have found that LRP2 could faciliate the endocytosis of Aβ as well as its clearance through blood cerebrospinal fluid barrier and blood-brain barrier [46, 52].

After binding of LRP2 to clusterin, the clearance of Aβ by LRP2 in BBB increases, indicating that the interaction between LRP2 and clusterin promotes Aβ efflux (Fig. 1) [53]. Further researchers observed that the efflux of clusterin increased when complexed to Aβ40 in vitro BBB model [54], so clusterin is important for the modulation of Aβ40 transcytosis across the BBB. Carro [55] found that selective deletion of LRP2 in the brain capillary endothelial cells of mice could originate behavioral impairments and neurodegeneration, which were the common clinical manifestations and pathological changes seen in AD brains. However there was no increase in the Aβ laden in the LRP2 deletion model and further studies are required to clarify the role of LRP2 in Aβ clearance. Serum insulin-like growth factor I (IGF-I) is neuroprotective, and in choroid plexus LRP2 can mediate the IGF-I-induced clearance of Aβ and promote the transport of IGF-I into the brain. So LRP2 is able to facilitate Aβ clearance and inhibit tau phosphorylation or amyloid neurotoxicity through mediating transport of IGF-1 [46].

#### Other receptors mediated Aβ efflux

As mentioned above many studies have proved that LRP and P-gp participate in the clearance of Aβ, however some researchers found that these two receptors do not play a major role in Aβ_40_ clearance by BBB. [56] So there must be other molecules involved in Aβ_40_ transport through BBB. It has been found that insulin can significantly inhibit [125I] Aβ_40_ through BBB in rats, whereas insulin receptor-specific inhibitors cannot block the elimination of [125I] Aβ_40_ across BBB into blood, [57] which suggests that there are unknown insulin-sensitive receptors involved in the elimination of [125I] Aβ_40_.

Natriuretic peptide receptor C (Npr-C), expressed in the brain capillary endothelium, can mediate the elimination of atrial natriuretic peptide (ANP) across BBB. Ito [58] found that ANP elimination can be inhibited by Aβ_40_, however, there was no direct interaction between Npr-C and Aβ_40_, indicating that the clearance of Aβ_40_ may be facilitated by other ANP-sensitive receptor expressed in cerebrovascular endothelium. Meanwhile in vitro study they found that insulin-degrading enzyme was involved in Aβ_40_ clearance through insulin-sensitive transporter and ANP-sensitive receptor in addition to the direct degradation of the protein. As a result, high ANP level caused by cardio-cerebrovascular disease in the brain may suppress the transport of Aβ across BBB to some extent, which could aggravate Aβ-induced pathological changes. However the structure and the function of these receptors are not clear and more studies are required to elucidate their role in blood brain barrier and Aβ clearance.

### Interstitial fluid bulk-flow clearance

#### Perivascular drainage

**Fig. 2:**
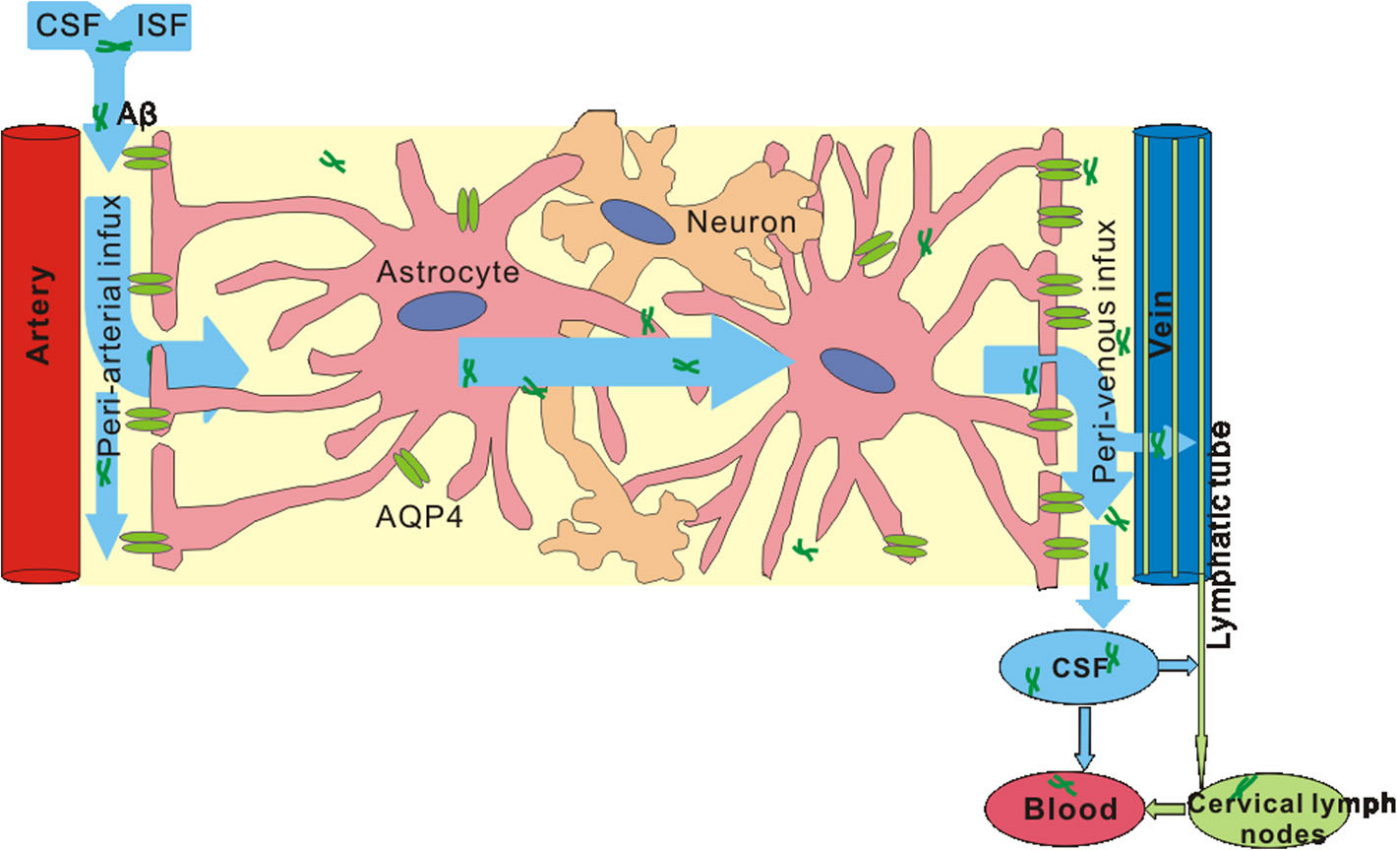
Brain glymphatic pathway facilitates the drainage of excess Aβ in CSF and ISF. CSF can flows into perivascular space through Virchow-Robin space, and then enters into brain parenchyma mixing with extracellular ISF. The CSF and ISF can travel along the arterial and capillary membrane then flow into leptomeningeal blood vessels or subarachnoid space, or move to cervical lymphnodes by lymphatic drainage and finally flow into blood. The higher expression of AQP4 surrounding veins provides an arteriovenous hydrostatic gradient to drive glymphatic drainage

After released from the neuron, Aβ_42_ tends to aggregate and form parenchyma senile plaques, but Aβ_40_ is resistant to aggregation in parenchyma and can be removed through the drainage of interstitial fluid along the cerebral capillaries and arteries (Fig. 2), where part of the protein can be eliminated through BBB (Figure 1). Any dysfunction in this process can trigger or promote the accumulation of Aβ_40_ in the vascular basement membrane leading to CAA pathological changes [59].

#### Vascular basement membrane

Vascular basement membrane is a special extracellular matrix of the endothelial basal surface, mainly secreted by endothelial cells. The main components include type IV collagen, laminin, nestin, heparan sulfate proteoglycan and other molecules. Cerebrovascular basement membrane plays a key role in vascular development, formation, BBB maintenance, and migration of peripheral cells including leukocytes [60]. There is continuity between brain extracellular matrix and cerebral capillary basement membrane (Fig. 1). So it is possible that interstitial fluid may be drained through the perivascular pathway to the peripheral which includes Aβ.

Increased age-related risk factors for cerebrovascular, such as arteriosclerosis, are also the risk factors for CAA and AD. Further study found that cerebrovascular basement membrane thickening, vacuolization, reduplication appeared in the elderly and the old mouse, were more predominant in the AD brain [61]. As to the workforce of the drainage system, it has been found that the drainage is only present in the live animals and it would stop when there is a cardiac arrest. So it is the blood flow that provides the main impetus for perivascular drainage [62]. After each heart beat the blood vessel will produce a reverse wave with the opposite direction of the blood flow, which appears to facilitate fluid and solute transport in perivascular drainage [63]. As a result, age-related cerebrovascular sclerosis, fibrosis, and loss of smooth muscle cells may reduce the artery contractile force, which leads to the weakness of the perivascular drainage and then induces the impairment of Aβ clearance and increases CAA risk.

#### Components’ changes of cerebrovascular basement membrane

CAA transgenic mouse show a significant reduction in type IV collagen, laminin, nidogen and an obvious increase in heparan sulfate proteoglycan compared to wild type mouse. More importantly the morphological and functional effects of aging on cerebral basement membranes and perivascular drainage differ between brain regions, with a more obvious influence on hippocampus [61]. As a risk factor for sporadic AD and CAA, ApoE4 has been shown to interfere with perivascular drainage of soluble Aβ, which may be achieved by the alteration of protein expression in the vascular basement membrane [64].

#### The role of apolipoprotein in perivascular drainage

ApoE protein in the cerebral vascular wall increased after anti-amyloid β immunotherapy [65], and study has suggested the co-localization of ApoE and Aβ in the perivascular drainage route (Fig. 1) [66]. After intraventricular injection of Aβ_40_ in ApoE transgenic mice, researchers found the co-deposition of ApoE4 and Aβ_40_ in the vessel wall rather than ApoE3 [64], indicating that the drainage rate of Aβ40 mediated by ApoE4 is much slower. Probably because the binding force between ApoE4-Aβ complex and basement membrane laminin is much weaker than that of ApoE3-Aβ complex, which suggests the impaired clearance of ApoE4-Aβ complex through perivascular drain compared to other ApoE-Aβ complexes [67]. ApoE4 has lower antioxidant activity than other ApoE isoforms [68] thus accelerate the loss of vascular integrity, breakdown of BBB which contributes to CAA. Another apolipoprotein clusterin was found to have high immunoreactivity in the arterioles and capillaries of AD and CAA patients, indicating clusterin is more likely to co-locate with Aβ40 rather than Aβ42 [69], so that clusterin might mediate the elimination of Aβ40 through perivascular drainage pathway.

#### Association between brain parenchymal and cerebrovascular Aβ deposition

Passive immunotherapy against Aβ in AD mice model and human has been confirmed to reduce amyloid deposition but it aggravates CAA pathological lesions [70]. Aβ deposition on the cerebral vessels flowing passive immunotherapy contains more Aβ_42_, which suggests that the parenchymal insoluble Aβ can be transformed into soluble form by specific antibody and then transported into the vascular basement membrane, and the failure of periarterial drainage aggregates CAA in AD patients.

Diem et al. established a computational model to investigate the Aβ periarterial drainage in the context of diffusion in the brain, and their studies showed that periarterial drainage of Aβ along basal membranes was more rapid compared with diffusion [71]. These results demonstrate that periarterial drainage is involved in the pathogenesis of CAA and AD as well as immunotherapy related complications. Meanwhile this indicates, to some extent, that normal cerebrovascular function is critical to the success of AD immunotherapy. For example, vasoactive drug cilostazol, a selective inhibitor of phosphodiesterase (PDE) III, has recently shown to significantly improve cognitive decline in patients with mild cognitive impairment [72]. Further animal study revealed that cilostazol reduced Aβ_40_ deposits and rescued cognitive decline in Tg-SwDI mice by promoting perivascular drainage of soluble Aβ_40_ [7].

Therefore, the composition, protein expression and morphology changes of the cerebrovascular basement membrane can impair the perivascular drainage of Aβ, and the deposition of Aβ on the vessel wall can further aggravating Aβ drainage obstacles. This vicious cycle may be an important precipating factor for CAA pathology. Understanding the dynamics of perivascular drainage of the brain will help to find new therapeutic intervention for CAA and AD.

### Glymphatic pathways

The glymphatic system is named based on its functional similarity to the peripheral lymphatic system, acting as a convective flux of CSF and ISF in the brain and strictly dependent on water channel aquaporin-4 (AQP4) expressed on the perivascular astrocytic endfeet [73]. Using in vivo two-photon microscopy in mice the dynamics of the glymphatic pathway was described for the first time. ISF solutes diffuse and finally enter the capillary and arterial basement membrane [74], then flow to the leptomeningeal blood vessels at the surface of the brain and finally move to cervical lymph nodes. CSF can flow into perivascular drainage route through Virchow-Robin space, and then enter into brain parenchyma mixing with extracellular ISF (Fig. 2) [75]. The perivascular drainage pathway was considered to be the lymphatic drainage in the brain, and it’s still unclear whether these two pathways are distinct pathways or perhaps they just reflect the same transport pathway under different physiological or experimental conditions [36].

Recent animal studies found that AQP4-dependent glymphatic pathway played an important role in promoting clearance of soluble Aβ from CSF and extracellular fluid. In mouse lacking AQP4 in astrocytes, the Aβ clearance was reduced by 55–65% compared with wild mouse. And the expression of AQP4 surrounding veins is higher than arterial, perhaps providing an arteriovenous hydrostatic gradient in order to drive perivascular CSF and ISF bulk flow [76]. In aging mouse brain loss of perivascular AQP4 results in impairment of perivascular CSF recirculation and Aβ clearance [77]. And deletion of AQP4 in AD transgenic mice aggregates brain Aβ accumulation and memory impairment [78]. In postmortem human tissue studies AQP4 was shown to be abnormally expressed in AD and CAA brains [79], and loss of perivascular AQP4 localization was associated with AD status [80]. This is intriguing, for reason that AD is associated with reactive gliosis. We speculate that altered AQP4 expression and depolarization in perivascular astrocytic endfeet under neuropathological conditions may be a triggering factor that contribute to impaired interstitial bulk flow and renders the aging brain more vulnerable to Aβ accumulation in vessel walls. That needs more studies to clarify the internal mechanism.

Following the clearance of free Aβ from the brain ISF into CSF sink via bulk flow, the proteins must be removed into the circulation or possibly through the meningeal lymphatics into the lymphatic system (Fig. 2) [81]. In peripheral organs ISF drains from tissues to lymph nodes. Previously it was thought that the central nervous system (CNS) lacked lymphatic vessels. A recent study has discovered the meningeal lymphatic vessels which expressed the specific molecular markers of lymphatic endothelial cells and are able to drain CSF and ISF into the deep cervical lymph nodes [81]. So after the clearance of Aβ through ISF-to-CSF bulk flow these meningeal lymphatic vessels may provide a conventional path for its further elimination to the peripheral. As discussed above, cilostazol can improve cognitive function of MCI patients [82] and the protective effect is probably not only related to its antiplatelet and vasodilator ability, we suspect that its ability to improve lymphatic function [83] may also be involved, although this hypothesis needs further studies to improve.

### Complement-related clearance system

**Fig. 3:**
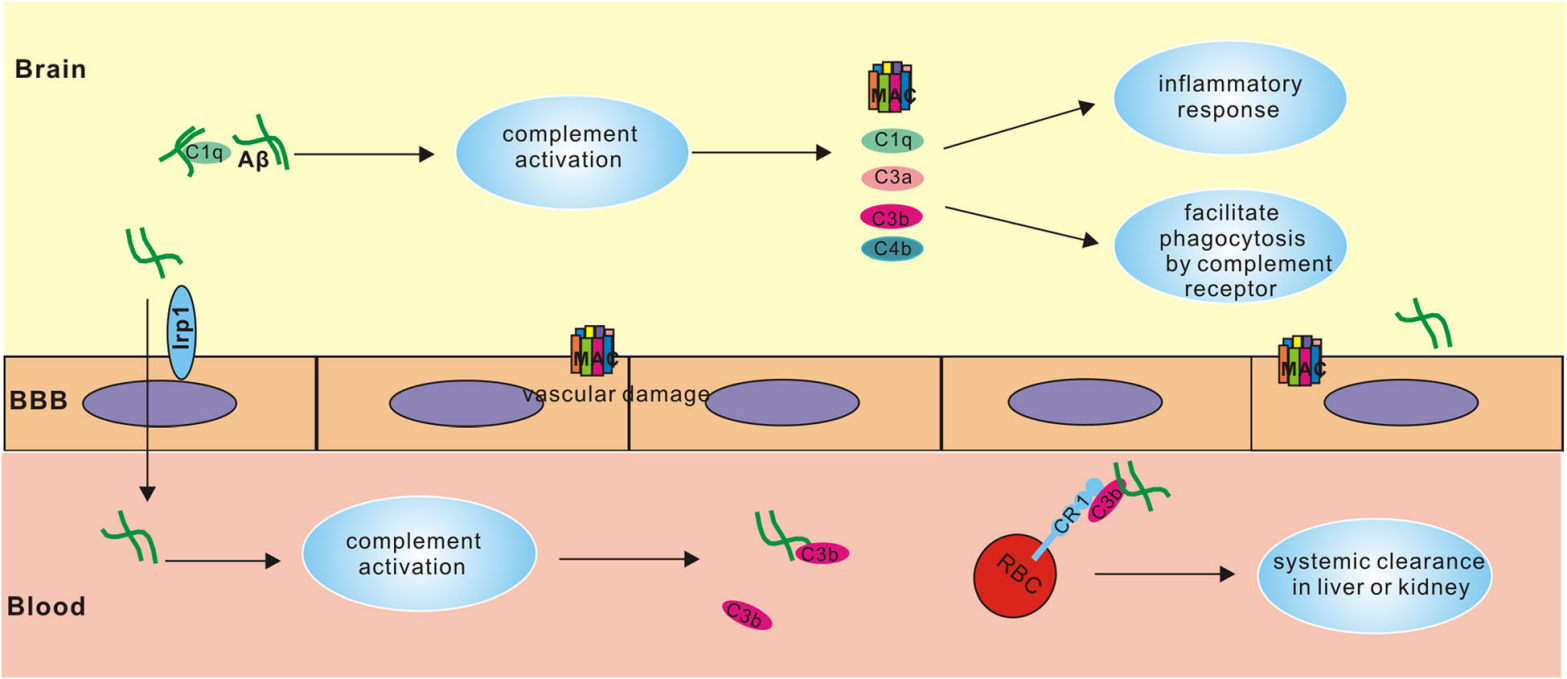
Complement activation plays both protective and detrimental roles in CAA and AD. Complement activation caused by Aβ can trigger inflammatory response in CNS, and the released inflammatory mediators together with the formation of MAC complex further leads to neuronal and vascular damage. However in CNS complement activation components can bind to Aβ-induced apoptotic cell surface and assist their phagocytosis by microglia which expresses many complement receptors. And in peripheral bloodstream Aβ can be transported by CR1 on erythrocyte after binding to C3b and further cleared in liver or kidney

Brain inflammation commonly occurs in CAA and AD, in which the accumulation of Aβ in the arterioles and capillaries of CAA patients might activate the complement system. The activated complement components, as a consequence, can produce a chronic, cumulating and low-level inflammatory response during disease course [84]. Aβ binding to C1q can activate complement system by classical pathway or by alternative pathway without C1q [85] (Fig. 3). These complement components are mainly expressed by neuron, microglial, astrocyte and cerebral microvascular endothelial cells (CMEC). Further study showed that complement proteins can also be produced by cerebrovascular smooth muscle cells and their activation aggravates vascular damage [86]. A recent study revealed that C3 secreted by astrocyte can interact with C3aR in microglial, mediating the inflammatory response induced by Aβ in central nervous system. Aβ can up-regulate the expression of NF-κB in astrocyte and promote the complement activation. Moreover, Aβ attenuated phagocytosis opsonized by complement and resulted in cognitive decline and Aβ deposition. C3aR antagonist can improve glial cell hyperplasia and Aβ deposition, exerting a therapeutic effect on the chronic inflammation of the nervous system [87]. However brain Aβ deposition increased when the activation of C3 was inhibited by expressing soluble complement receptor- related protein y (sCrry) in human amyloid precursor (hAPP) transgenic mice [88]. Complement activation components bind to Aβ-induced apoptotic cell surface, and the microglia complement receptors are implicated in their clearance [89]. So certain inflammatory response in the brain appears to be favorable to neurodegenerative disease.

CR1, also known as CD35 or C3b/C, is an impotent protein in the complement regulatory system, which can enhance the endocytosis of C3b, C4b, C1q coated particles by phagocytic cells and regulate complement activation by inhibiting C3 and C5 invertase activity. Immune complexes, aberrant antibodies or Aβ can be transported by erythrocyte CR1 receptors to the liver for removal. Rogers found that Aβ can be cleared from the bloodstream by CR1 receptors on erythrocyte after binding to C3b (Fig. 3) [90]. In addition, AD and MCI patients show lower levels of C3-opsonized erythrocyte Aβ compared to cognitive normal subjects. So CR1 may be involved in Aβ metabolism and the dysfunction of C3b-dependent Aβ adherence to CR1 can increase the risk of AD. However, it remained unclear how this additional C3b binding site of CR1 exerts its risk for CAA and AD.

In recent years, genome-wide association mapping studies (GWAS) have provided strong evidence for CR1 single nucleotide polymorphism (SNP) being a risk factor for sporadic AD, in which SNP rs6656401 was positively correlated with Aβ deposition in brain [91, 92, 93]. Given the overlap of CAA and AD pathological features, Biffi investigated whether this variant is also correlated with CAA risk and its pathological changes [94]. They found that rs6656401 aggravated CAA-related intracerebral hemorrhage (ICH), risk of recurrent CAA-ICH, as well as cerebrovascular Aβ deposition. So complement system may be an evoking factor for the pathogenesis of AD and CAA, not just being activated as a result of Aβ deposition. Rs6656401 is located on the non-coding region of CR1 gene and CR1 gene containing this SNP encodes CR1 protein with additional C3b binding sites [95]. It is possible that these additional C3b binding sites of CR1 increase the ability of CR1 to carry more Aβ for liver to degradation.

The only SNP found in the coding region of CR1 gene so far is rs4844609 which encodes a domain that binds to mannan-binding lectin (MBL) and fibronectin [96]. MBL are found to bind to Aβ [97], however the relationship between MBL and AD as well as CAA is unknown. Since C1q, C4b, and C3b are associated with Aβ plaques, it is speculated that rs4844609 and rs665401 may influence the affinity of CR1 with C1q, C4b, and C3b by altering the molecular structure of the receptor and further affect the clearance of Aβ. Rs4844609 can also regulate the cleavage site of the CR1 protein to produce soluble CR1 (sCR1) [98]. sCR1 could regulate complement activation on cell surface and may affect the removal of immune complex and Aβ. A recent study revealed that there is a slight difference in sCR1 levels among populations with different CR1 genotype, and CR1 has a better affinity for C1q and C3b in people with rs4844609, but these differences are not associated with changes in cognitive function [99]. Additionally, it’s unknown whether different CR1 isoforms can influence the clearance of peripheral Aβ. So more precise methods are needed to determine the affinity of different CR1 isoforms with C1q, C3b and to identity the molecular pathways of these polymorphisms as related to AD or CAA susceptibility.

## Conclusions

The aggregation of Aβ in the brain increases with aging, so age-related risk factors are also associated with impaired Aβ metabolism. As a result the incidence of CAA and AD significantly increases in the aging population, bringing great burden to society and their families. The deposition of Aβ on the vessel wall can induce the release of inflammatory factors, complement activation, oxidative stress, which further lead to the damage of vascular endothelium and smooth muscle cells resulting in intracerebral hemorrhage, ischemic stroke as well as cognitive decline. However, until now there’s no effective way to prevent or reverse CAA pathological changes and the clearance of Aβ is regulated by variable factors and many of them are still unclear. So identification of molecular pathways involved in Aβ clearance will provide new targets for AD and CAA therapeutic intervention.

## Abbreviations

ABC: ATP-binding cassette
AD: Alzheimer’s disease
ANP: Atrial natriuretic peptide
APP: Amyloid precursor
Aβ: Amyloid β-protein
BBB: Blood brain barrier
BCRP: Breast cancer resistance protein
BCSFB: Blood–cerebrospinal fluid barrier
CAA: Cerebral amyloid angiopathy
CEMC: Cerebral microvascular endothelial cell
Crry: Complement receptor- related protein y
CSF: cerebral spinal fluid
ET-1: Endothelin-1
GWAS: Genome-wide association mapping studies
ICH: Intracerebral hemorrhage
IDE: Insulin-degrading enzyme
ISF: Interstitial fluid
LDLR: Low-density lipoprotein receptor
MBL: Mannan-binding lectin
MDR1: Multidrug resistance proteins
MRPs: Multidrug resistance-associated protein
Npr-C: Natriuretic peptide receptor
OATP: Organic anion transporting polypeptides
PDE: Phosphodiesterase
P-gp: P-glycoprotein
RAGE: Advanced glycation end products
SRF: Serum response factor
α2M: α2-microglobulin
